# Comparison of estimated energy intake using Web-based Dietary Assessment Software with accelerometer-determined energy expenditure in children

**DOI:** 10.3402/fnr.v57i0.21434

**Published:** 2013-12-17

**Authors:** Anja Biltoft-Jensen, Mads F. Hjorth, Ellen Trolle, Tue Christensen, Per B. Brockhoff, Lene F. Andersen, Inge Tetens, Jeppe Matthiessen

**Affiliations:** 1 Department of Nutrition, National Food Institute, Technical University of Denmark, Søborg, Denmark; 2 Department of Human Nutrition, Faculty of Life Sciences, University of Copenhagen, Frederiksberg C, Denmark; 3 Department of Applied Mathematics and Computer Science, Technical University of Denmark, Kongens Lyngby, Denmark; 4 Department of Nutrition, Institute of Basic Medical Sciences, University of Oslo, Oslo, Norway

**Keywords:** children, reporting accuracy, under-reporting, over-reporting, food diary

## Abstract

**Background:**

The OPUS (Optimal well-being, development and health for Danish children through a healthy New Nordic Diet) project carried out a school meal study to assess the impact of a New Nordic Diet (NND). The random controlled trial involved 834 children aged 8–11 in nine local authority schools in Denmark. Dietary assessment was carried out using a program known as WebDASC (Web-based Dietary Assessment Software for Children) to collect data from the children.

**Objective:**

To compare the energy intake (EI) of schoolchildren aged 8–11 estimated using the WebDASC system against the total energy expenditure (TEE) as derived from accelerometers worn by the children during the same period. A second objective was to evaluate the WebDASC's usability.

**Design:**

Eighty-one schoolchildren took part in what was the pilot study for the OPUS project, and they recorded their total diet using WebDASC and wore an accelerometer for two periods of seven consecutive days: at baseline, when they ate their usual packed lunches and at intervention when they were served the NND. EI was estimated using WebDASC, and TEE was calculated from accelerometer-derived activity energy expenditure, basal metabolic rate, and diet-induced thermogenesis. WebDASC's usability was assessed using a questionnaire. Parents could help their children record their diet and answer the questionnaire.

**Results:**

Evaluated against TEE as derived from the accelerometers worn at the same time, the WebDASC performed just as well as other traditional methods of collecting dietary data and proved both effective and acceptable with children aged 8–11, even with perhaps less familiar foods of the NND.

**Conclusions:**

WebDASC is a useful method that provided a reasonably accurate measure of EI at group level when compared to TEE derived from accelerometer-determined physical activity in children. WebDASC will benefit future research in this area.

Valid and reliable dietary assessment methods are critical for identifying the impact of diet interventions on children's dietary habits and their health and weight status, and for the future development of successful prevention and intervention strategies.

Dietary habits are probably established already in childhood and become increasingly rooted in adolescence and adulthood (1). Consequently, it can be expected that healthy eating habits acquired early in life will have a higher chance of being maintained, reducing the risk of obesity and other lifestyle diseases later in life. A number of intervention studies have been conducted in schools with the purpose of changing unhealthy eating habits among children (2, 3). The advantage of using a school-based approach is that children attending local authority schools constitute a population with a mixed ethnic and socioeconomic background. Here, it is possible to reach all children, including socially disadvantaged children, who are likely to benefit most from such a healthy diet intervention.

The OPUS (Optimal well-being, development and health for Danish children through a healthy New Nordic Diet) Centre was established in 2009 to advance public health and prevent obesity among children. OPUS promotes the concept of the New Nordic Diet (NND), which draws on sustainable food items, such as whole-grain, fruits and berries, root vegetables, cabbages, legumes, game, seaweed, fish, and nuts, native to the Scandinavian region. The NND is described in more detail elsewhere (4, 5). The OPUS School Meal Study used these foods in the school lunch menus and snacks of 834 schoolchildren aged 8–11, to investigate the effect of the NND on BMI, body composition, sleep, and risk markers for lifestyle diseases (5).

To measure the children's intake of the NND and the impact of the NND on the children's normal diet, an appropriate dietary assessment tool was needed. The diet reporting presented several challenges: Recipes and meals could change at the ‘last minute’ due to the changing availability of ingredients, and some foods and dishes, for example, seaweed, cabbage, and legumes, might be unfamiliar to both child and parents. We considered that an interactive and web-based, self-administered seven-day food diary or recall method would be feasible for reporting the NND lunch and snacks, flexible enough to cope with changing foods and recipes, and cost effective to use for this study (6). Computer-, PDA-, or mobile-phone-assisted questionnaires are becoming increasingly common (7–13). Using such web-based technology to collect dietary intake data offers the opportunity to make an appealing interface that is especially engaging to children, adolescents, and younger adults, who are familiar with the technology in their daily lives. Computerized diet programmes get high ratings as ‘enjoyable’ and ‘easy to use’ (14). A number of web-based methods have proved both feasible and acceptable when used with children (7, 9, 15–17).

The Web-based Dietary Assessment Software for Children (WebDASC) was developed for the purpose of assessing dietary intake among children aged 8–11 in the OPUS School Meal study and in intervention studies in general (18). As far as the authors know, this is the first such system developed and evaluated in Scandinavia.

To evaluate the accuracy of the WebDASC, we needed an objective measure to ensure that the dietary assessment instrument does not introduce errors that distort the true relationship between dietary intake and health. Data on a person's total energy expenditure (TEE) can be used to estimate any under- and over-reporting of energy intake (EI) in conditions of energy balance. The gold standard reference method for validation of EI, double labelling, requires urine samples and is expensive in terms of both administration and analysis, and this was not an option in the present study. However, prediction equations to derive energy expenditure from accelerometer output together with basal metabolic rate (BMR) and diet-induced thermogenesis (DIT) can serve as a feasible and cost-effective method for validating recorded EI data (19). TEE derived from motion monitors has previously been used to evaluate EI in children and adults (20–25). Furthermore, accelerometer measurements can also be used for estimating physical activity and sleep – two other central measures of the OPUS study. Consequently, accelerometry seemed a good and feasible choice as a reference method.

This article presents the comparison of WebDASC-reported EI against TEE derived from accelerometers on schoolchildren aged 8–11 during two periods of seven consecutive days: at baseline when the children ate their usual packed lunches and at intervention when children were served NND for school lunch and snacks. We also present an evaluation of the usability of WebDASC.

## Experimental methods

### Study design

**Fig. 1:**
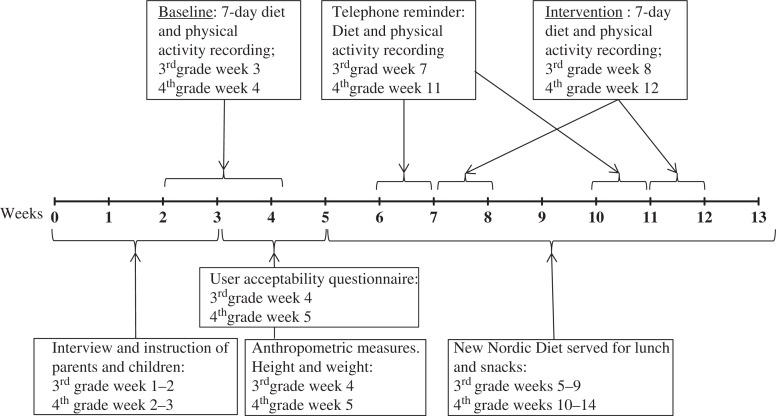
Design of the Web-based Dietary Assessment Software for Children validation study.

The research was a part of the OPUS School Meal pilot study, which was conducted to test multiple measurement procedures, logistics, cooking, and serving of NND meals for schoolchildren. The data collection was performed in January (baseline) and in February/March (intervention) 2011. The full pilot study design is illustrated in Fig. 1. The schoolchildren had their usual packed lunches during the baseline period and were served the NND, including a mid-morning snack, an *ad-libitum* hot lunch meal, and an afternoon snack, during the intervention period. The menu plan for the NND served can be seen in the Appendix. The children, assisted by parents, recorded their diet in the evening using the WebDASC during each of the time periods of seven consecutive days: baseline and intervention. In the same periods, the children wore accelerometers. Detailed oral and written instructions on how to record food intake and how to use the accelerometer were given individually to all the children and their parents. In addition, trained personnel interviewed parents on their social background, health issues, and attitudes and knowledge about food and health. After the baseline assessment, participants completed a user acceptability questionnaire about the WebDASC, and trained personnel in the OPUS mobile laboratory took anthropometric measurements of body weight and height.

This study was conducted in accordance with the guidelines laid down in the Declaration of Helsinki and the Biomedical Research Ethics in the Capital Region of Denmark approved all procedures involving human subjects.

### Participants

Children in 3rd and 4th grade at a school in north-eastern Denmark, in total 105 pupils aged 8–11, and their families were invited to take part, and 81 gave their written informed consent.

### WebDASC

WebDASC was developed as an interactive food record–recall method. Participants recorded their diet in WebDASC in the evening after the final eating occasion on each day for seven consecutive days.

WebDASC guides respondents through six daily eating occasions (breakfast, morning snack, lunch, afternoon snack, dinner, and evening snack). For the diet recording, a database of 1,300 food items was available, either through category browsing or free text search, aided by a spell-check application. It was possible to type in foods not otherwise found through category browsing or text search. The amount consumed was estimated by selecting the portion size from four different digital images among 320 photo series. Furthermore, participants recorded any intake of supplements, whether a recording day represented usual or unusual intake, and reasons for unusual intakes, such as illness.

WebDASC includes internal checks for frequently forgotten foods (spreads, sugar, sauces, dressings, snacks, candy, and beverages).

**Fig. 2:**
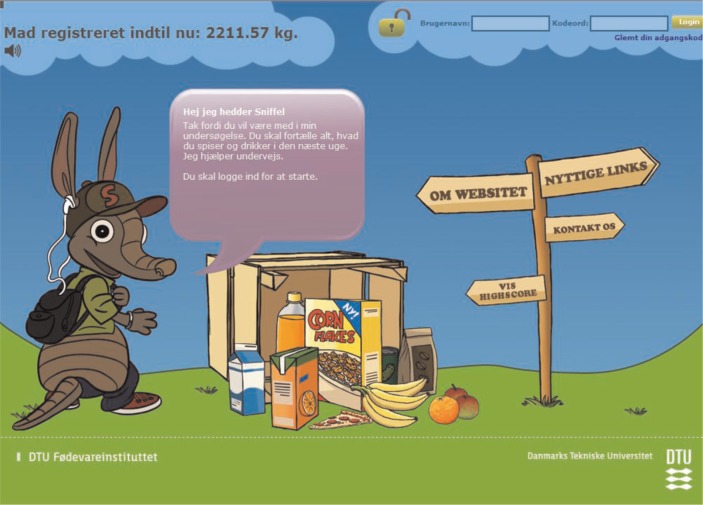
WebDASC opening screen.

**Fig. 3:**
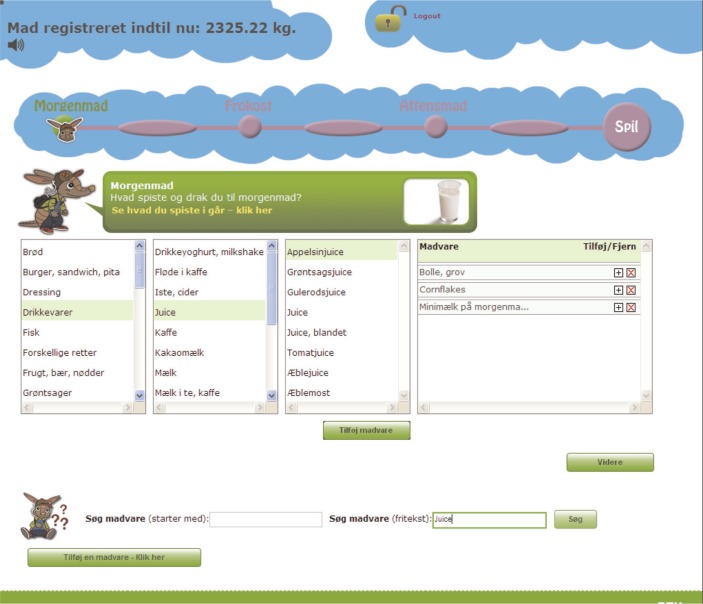
WebDASC food search and selection screen.

To make the interface appealing for children, WebDASC uses an animated armadillo as a guide and the following features to create motivation: a food-meter displaying the total amount of food recorded so far, a most-popular-food ranking, and a computer game with a high score list. The rank list and game is accessible after completing one recording day. The opening screen and food search and selection screen are illustrated in Figs. 2 and 3. If a participant failed to report food intake one day, parents were reminded the next day by e-mail. If a participant failed to record food intake for a day within 48 h, the recording day was automatically closed for further registration, and the next recording day was opened.

For participants to be included in the analyses, the WebDASC had to be completed for at least three weekdays and one weekend day. The EI was calculated for each individual using the software system GIES (Version 1.000 d – 2010-02-26) developed at the National Food Institute, Technical University of Denmark, and the Danish Food Composition Databank (version 7; Søborg; Denmark; 02-03-2009).

### Accelerometry

The children were instructed to wear the accelerometer (ActiGraph^TM^ GT3X, Tri-Axis Accelerometer Monitor, Pensacola, FL) 24 h a day for seven consecutive days. The accelerometer was worn in an elastic belt on the right hip, also when asleep, and the participants were instructed to remove it only during activities involving water, as when showering or swimming. The freely available software Propero Actigraph analysis software (version 1.0.18; http://sourceforge.net/projects/propero/files/) was used to analyse the accelerometer data. As recommended by Treuth and colleagues (26), time periods of at least 20 consecutive minutes of zero counts were considered as representing periods when the monitor was not worn or when the child was sleeping and were thus disregarded before analysis. Derived variables were mean counts per min (cpm) based on the vertical axis. Criteria for a successful recording were a minimum of four days of 13 h wear-time per day including at least three weekdays and one weekend day as for the diet assessment.

Activity energy expenditure (AEE) was derived from the mean cpm using Ekelund et al.'s modified prediction equation (27): AEE (kcal/day) = 66.847+(cpm×0.953)–(176.91×gender). TEE was then calculated as AEE plus BMR plus DIT, which was assumed to account for 10% of TEE (28) (TEE = AEE + BMR + DIT). BMR was calculated using the Schofield equation as improved by Henry (29) based on age, sex, height, and weight.

### Anthropometric measurements

After the baseline diet and activity reporting, and after fasting overnight, participants were weighed once, without shoes and in light indoor clothing, to the nearest 0.1 kg on an electronic digital scale (Tanita BWB-800S, Tokyo, Japan). Their height was measured without shoes to the nearest 0.1 cm with a stadiometer (CMS Weighing Equipment LTD, London, UK).

### Usability

At the personal interview, each child was given a questionnaire to be completed with the assistance from one parent after the baseline recordings and returned to give us qualitative feedback on the user acceptability of the WebDASC. The questionnaire contained 18 questions, with 12 of the questions using rating scales, and the rest using either multiple choice or open responses. The questions included the amount of help provided by parents, the time spent recording information on the first day and the following days, how acceptable this time was, the preferred search functionality for the child and for the parents, how helpful the images of portion sizes were when estimating portion sizes, the usefulness of the guidance given, self-assessed reactivity, and questions about the interface design, the game, and suggestions for improvements.

### Statistical method

Our definition of under-, acceptable, and over-reporters of recorded EI was assessed using the confidence limits of agreement between the EI and TEE recorded at the individual level as suggested by Black (30). This method has been found suitable for adults. As far as the authors are aware, no cut-off methods specifically designed for children have been published. Including data from both measurement periods, we defined misreporters of EI using the ratio EI:TEE, and acceptable reporters were defined as having a ratio of EI:TEE in the range 78–122%. We defined under-reporters as having EI:TEE < 78% and over-reporters as having EI:TEE > 122%. Differences in the number of under-, acceptable, and over-reporters between baseline and intervention were evaluated using χ^2^ test.

The agreement between EI and TEE at group level was evaluated by comparing means, using the paired-sample *t*-test. Limits of agreement and distribution of bias over the range of energy values were assessed using a modified Bland–Altman plot for repeated measurements, which takes into account both a random error within and between individuals (31), which makes it possible to include each subject twice. The limits of agreement were defined as twice the corrected standard deviations of the differences above and below the mean.

The agreement between EI and TEE at the individual level was evaluated using the cross-classification of EI and TEE divided into quartiles and applying kappa statistics. Pearson's correlation coefficients were also calculated.

The repeatability of EI between baseline and intervention was assessed using the Intra Class Correlation Coefficient (ICC).

Linear-mixed models were used to assess a potential intervention effect (the period factor: baseline; intervention), the effects of gender, parental education, BMI, age, illness reported as affecting eating, and the mutual interactions of all these on EI:TEE. The fixed-factor effects in the model were gender, parental education, BMI, age, illness that affected eating, and measurement period, and their two-way interactions. To adjust for dependency in repeated measures within subjects, random effects were added for subject. The homogeneity of variance and normality of the residuals were examined using graphical methods.

In all the statistical analyses, a significance level of 5% was applied. Data were analysed using SPSS for Windows version 19.

## Results

The study population consisted of 81 children (34 boys; 47 girls), with a mean age of 10.3 years. The majority (54%) had parents with a vocational education. Ten percent of the study population was classified as overweight/obese according to the international age- and gender-specific child BMI cut-off points (32) (Table 1). All 81 children, assisted by parents, recorded their diet in the WebDASC and wore the accelerometer for at least five days or more in the WebDASC at baseline. At intervention, 78 had acceptable food recording and 73 had acceptable accelerometer results, which meant that 72 children (28 boys; 44 girls) had measurement data for both EI and TEE for the intervention period. Ninety-four percent had complete data for all seven days at baseline and 90% at intervention.

**Table 1:** Characteristics of the WebDASC* validation study sample

	WebDASC validation study sample (*n*=81)	
	
	Mean	SD
Subjects		
Boys/girls (%)	42/58	
Age (years)	10.3	0.6
Parental education (%)		
Basic school	4	
Vocational education (11–13 years, practical)	54	
Short further education (11–13 years, theoretical)	9	
Medium and long further education (>15 years)	33	
Weight (kg)	35.5	6.9
Height (cm)	144.0	7.2
BMI (kg/m^2^)	17.0	2.4
Overweight/obese† (%)	9/1	

* Web-based Dietary Assessment Software for Children.

† Overweight/obese is defined according to the international age- and gender-specific child BMI cut-off points (32).

### Agreement between EI and TEE

**Fig. 4:**
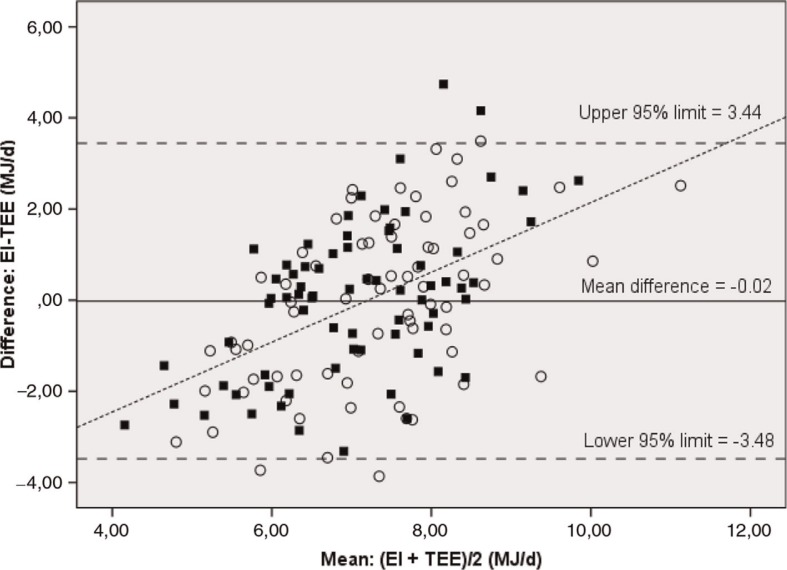
Bland–Altman plot for repeated measures of the differences between energy intake (EI) derived from the WebDASC* and accelerometer-determined energy expenditure (TEE) plotted against the mean of EI and TEE (*n*=72) (31). ■=baseline and ○=intervention. *Web-based Dietary Assessment Software for Children.

At group level, we found no differences between EI and TEE at baseline (−0.02 MJ/d; *p*=0.92) or at intervention (−0.06 MJ/d; *p*=0.790) (Table 2). EI, EI:TEE, EI:BMR did not differ significantly at baseline and intervention (7.1 vs. 7.3; 1.0 vs. 1.0; 1.42 vs. 1.47; *p*>0.2). The Bland–Altman plot of repeated measures demonstrated positive differences between EI and TEE at higher values of EI and TEE and negative differences at lower values (Fig. 4). The 95% limits of agreement were 3.48 and 3.44 MJ/d for both periods.

**Table 2:** Difference between reported EI using WebDASC* and TEE at baseline and intervention

	EI (MJ/day)		TEE (MJ/day)			95% Confidence intervals (MJ/day)		
					
	Mean	SD	Mean	SD	Mean difference (MJ/day)	LL	UL	*p*
Baseline (*n*=81)	7.08	1.65	7.10	0.99	−0.02	−0.38	0.34	0.915
Intervention (*n*=72)	7.29	1.85	7.35	1.08	−0.06	−0.49	0.38	0.790
Baseline and intervention (*n*=153)	7.18	1.74	7.22	1.04	−0.04	−0.31	0.24	0.788

Paired sample *t*-test. EI = energy intake; TEE = accelerometer-derived energy expenditure.

* Web-based Dietary Assessment Software for Children.

Including both recording periods, the Pearson's correlation coefficient between EI and TEE was 0.31 (*p*<0.001). The proportion of participants appearing in the same or adjacent quartile for both EI and TEE was 73%; 20% was misclassified and 7% was grossly misclassified. The value for the Kappa coefficient was 0.128 (*p*=0.047) indicating slight agreement (33).

Separated by periods, the Pearson's correlation coefficient was 0.32 at baseline (*p*=0.004) and 0.30 at intervention (*p*=0.011). For the baseline period, 77% were classified into correct or adjacent quartiles and, for the intervention period, 69% were classified into correct or adjacent quartiles.

### Proportion of acceptable reporters and misreporters and their characteristics

Approximately 20% of the study population were defined as under-reporters and 20% as over-reporters of EI at baseline and intervention, reflecting the trend seen in the Bland–Altman plot. There was no significant (*p*=0.80) difference in the proportion of under-, acceptable, and over-reporters between baseline and intervention (data not shown). A significantly higher proportion of under-reporters were affected by illness than for acceptable and over-reporters (Table 3). More girls than boys were misreporters; under-reporters had significantly higher BMI compared to acceptable and over-reporters, while over-reporters had lower BMI compared to acceptable and under-reporters.

**Table 3:** Characteristics of under-, acceptable, and over-reporters of energy intake in the WebDASC* evaluation study (Baseline and intervention *n*=153)

	Under-reporters (*n*=33)		Acceptable reporters (*n*=89)		Over-reporters (*n*=31)	
			
	Mean	SD	Mean	SD	Mean	SD
Subjects						
Boys (%)	36^ab^		48^a^		23^b^	
Girls (%)	64^ab^		52^b^		77^a^	
Age (years)	10.4	0.5	10.3	0.6	10.2	0.6
Parental educational level (%)						
Basic school and vocational education (≤13 years mainly practical)	61		55		55	
Short, medium and long further education (>11 years mainly theoretical)	40		45		45	
Illness affected eating (%)	56^a^		27^b^		31^b^	
BMI (kg/m^2^)	18.6^a^	3.3	16.9^b^	1.9	15.4^c^	1.4
Overweight including obese† (%)	27 ^a^		6^b^		0	
BMR MJ/day	5.2^a^	0.5	5.1^a^	0.4	4.8^b^	0.4
Total energy expenditure MJ/day	7.4^a^	1.1	7.4^a,b^	1.0	6.7^c^	1.0
Energy intake MJ/day	5.0^c^	1.0	7.4^b^	1.1	9.0^a^	1.3
EI:TEE	0.7 ^c^	0.1	1.0^b^	0.1	1.4^a^	0.1

Statistical analysis included independent *t*-test and χ^2^ test.

Mean values within a row with unlike superscript lowercase letters were significantly different between under-, acceptable, and under-reporters (*p*<0.05).

* Web-based Dietary Assessment Software for Children.

† Overweight is defined according to the international age- and gender-specific child BMI cut-off points (32).

### Intervention effect and interactions with EI:TEE

The results from the linear-mixed models show that main effects of age, parental educational level, and measurement period (baseline vs. intervention) were not significantly associated with EI:TEE. Respondents who were not affected by illness had an 11% higher EI:TEE than those who were affected, mainly because of a higher EI (1,233 kJ/d higher). Boys had a 10% lower EI:TEE than girls, because they had a 1,682 kJ/d higher TEE. EI:TEE decreased 4% for every unit increase in BMI (Table 4).

**Table 4:** Association between background variables and EI:TEE (*n*=162)

	95% Confidence intervals				
	
Parameter	Estimate	SE	LL	UL	*p*
EI:TEE (%)					
Illness					
Not ill vs. illness ≥1 day	10.73	3.63	3.55	17.90	0.004
Gender					
Boys vs. girls	−9.96	4.44	−18.80	−1.12	0.028
BMI					
Increase per unit	−3.65	0.90	−5.44	−1.85	<0.001

### Repeatability

The ICC between baseline and intervention for EI was 0.45 (95% CI: 0.25–0.61) indicating moderate agreement.

### Usability

Seventy-four out of 81 who completed the dietary assessment at baseline returned the usability questionnaire. Ninety percent of the children received some help from parents to complete the WebDASC. The average time spent completing the WebDASC the first day was 35 min and 15 min on the following days. A total of 80% found the recording duration acceptable, and 85–90% found the task of the diet recording easy. Children preferred the browse search by category, whereas parents preferred the free text search. Both liked the user interface design.

## Discussion

Comparison of the WebDASC-estimated EI and the accelerometer-derived TEE indicated agreement at the group level. At individual level, the data showed substantial variation in accuracy. The ability of the WebDASC to rank individuals according to TEE was generally good and slightly better at baseline than at intervention. This difference may be due to difficulties in recording the NND, which included perhaps unfamiliar foods and dishes. Previous studies have shown that it is more difficult for children to record unfamiliar foods than familiar foods (34), so it is essential to make it easy to record the NND intervention foods in the WebDASC. Ideally, participants should have the daily menu beside them when recording so that they can accurately recall the names of the dishes. The daily menu was accessible in the WebDASC from the front page, but perhaps not everyone used this option.

As far as the authors are aware, no other studies have evaluated EI assessed by a seven-day Web-based dietary assessment method against an objective method for TEE. A few validation studies with children of similar age have used motion instruments as a reference method to validate paper versions of food records (24, 25). In these studies, EI was underestimated by 7–20% at group level in children aged 7–13 (7–20% (25); 18% (24)). In comparison, the present study showed less than 1% underestimation of EI at group level. At the individual level, the above-mentioned studies produced Pearson's correlation coefficients of 0.28 (24) and 0.29 (25) compared to 0.31 in the present study. Comparison of cross-classification and underreporting also showed similar results between the studies. Considering the challenges involved in recording the NND, it seems that the WebDASC performs just as well as other (paper-based) methods when compared with TEE derived from motion instruments.

Illness affected many children's eating during the recording periods – 27 and 14 reported that illness affected their dietary intake during the baseline and intervention period respectively, due to a flu epidemic. This may have affected the correlation analysis. Twenty-seven percent of the schoolchildren recorded a lower EI than their calculated BMR; half of these reported that they had eaten less than usual because of illness. Leaving out these individuals underreporting dropped to around 16%, which is similar to the findings in other studies (36). But using TEE as a reference measure for EI under these circumstances may be a problem, because BMR is a large component of TEE (70% of TEE in the present study). TEE will never be less than BMR, whereas EI can vary from a zero intake during illness and upwards. The wide variation in EI compared to the underestimated variation in TEE may have exaggerated the difference between EI and TEE. Correlations performed with the variable part of TEE, the AEE, produced a higher correlation of 0.4 overall. Compared to the TEE estimated from AEE, BMR, and DIT, the estimated EI therefore seemed to perform slightly worse in the current study than would be expected under normal circumstances.

### Misreporting

Approximately 20% were classified as over-reporters and 20% as under-reporters. This is different from other validation studies using motion sensors to validate EI in children, which argue that under-reporting is a large problem (23, 24, 35). Data from studies documenting over-reporting of EI in children are sparse. In the review of Forrestal (36), only two studies described substantial over-reporting in children aged 9 and 4–11, respectively. Using portion size images for portion size estimation of all foods and beverages recorded in the WebDASC may have made it easier to be consistent in either under- or over-reporting compared to other similar dietary assessment methods using a mixture of standard portions and portion size images (10, 24, 25).

In the present study, the children characterized as over-reporters had lower BMI compared to acceptable reporters and under-reporters. This was also observed in a study among children aged 4–11, in which over-reporters weighed less than under- and accurate reporters (37). These findings support the idea that children with a low BMI (or their parents) may be more likely to report larger portion-sizes as they wish (for their child) to increase growth and eat more. It has been shown that maternal pressure to eat is inversely correlated to children's BMI (38).

Under-reporters were more likely than both acceptable and over-reporters to report that illness affected eating during the recording periods. This was confirmed by the results from the linear-mixed models that showed that absence of illness influenced EI:TEE positively. This has also been reported in a study with adults, in which illness during the recording period had a significant impact on under-reporting (39). A German validation study with children and adolescents (1–18 years) found, in agreement with these findings, that acceptable reporters had a higher percentage of normal recording days (36).

Under-reporters had higher BMI and recorded less EI compared to acceptable and over-reporters. Other studies have also shown that under-reporters aged 7–11 have higher BMI and are more worried about weight than acceptable reporters and over-reporters (40–42). Recording accuracy may also be compromised by overweight parents underestimating their children's intake (42). Parents in the present study were more likely to be overweight than a representative sample of parents for the same age group in the Danish National Survey of Diet and Physical Activity (DANSDA) 2003–2008 (54% vs. 42%).

### Repeatability of recorded EI

The repeatability of EI between baseline and intervention was moderate. There were some limitations with the repeatability assessment, because the conditions between the baseline and intervention period differed with the NND served for school lunch and snacks in the intervention period. It seems reasonable to expect that reported EI should be approximately at the same level during the two periods since the intervention only covered school meals where food were offered *ad-libitum*. However, it may have been more difficult for the children and their parents to report the NND in the WebDASC than their usual packed lunches. This may be reflected in the moderate repeatability.

Due to limited resources and the design of the pilot study, the children were only weighed once (after the baseline reporting period). Energy balance could therefore not be confirmed in the present study. However, the dietary assessment period was too short for energy imbalance to present as notable weight change.

During growth and development children are normally in a positive energy balance, but energy accretion is about 1–2% of EI (34, 43), which would not influence the overall results.

### Strengths and limitations

One major strength in the present study lies in the use of a reference method to derive TEE, which do not have any errors correlated with the dietary assessment method, as would be a risk if another dietary assessment method was chosen as the reference. Other validation studies of web-based methods in connection with children have used relative validation methods (7, 9, 15). We have used an objective method, which has proved useful with both children and adults (19, 44).

The results from the qualitative questionnaire showed that the WebDASC method was well accepted by participants, who also provided useful feedback for improving the interactive recording method. The present study population consisted of a higher proportion of children whose parents have a vocational education than is the case in the general population (data not shown). This suggests that WebDASC works well, irrespective of parental educational level.

Moreover, we found no difference in recorded EI between measurement periods in the present study. This may be a result of the standardized WebDASC interface guiding respondents through all meals, the use of questions with a conditional response option, which makes it difficult to skip responses, and the use of probing and internal checks to enhance memory and food recording.

In general, the recorded level of EI is low compared to EI recorded by the same age group in the DANSDA 2003–2008 and compared to international reference values, but TEE was also low. This could be a seasonal effect because data were collected during the winter, whereas DANSDA data are collected throughout the year (45). Kolle et al. (46) also found seasonal variations in physical activity level where they used accelerometers in a representative sample of 9-year-old Norwegian children, with their physical activity level being lowest during the winter. Furthermore, a large number of children reported illness during both periods, due to a flu epidemic. As a result of the seasonal low activity level and the illness among children, we measured less variation in AEE than expected and AEE accounted for 20% of TEE.

### Accelerometer-determined TEE

Accelerometers do not accurately capture certain forms of activity, such as arm movement, carrying loads, and cycling, due to the way the instrument is designed. Moreover, the accelerometer was removed during water activities, such as swimming. However, the average duration of these activities was very low (14 min/day) in the present study, and cannot explain the low TEE.

Errors can have been introduced in estimating the different parts of TEE, because the choice of cut-off for non-wear time to derive AEE may have affected the number of misreporters in either direction. There is no consensus about the most appropriate energy expenditure prediction equation to use with regard to accelerometer data, including how to distinguish between periods of non-wear and bouts of sedentary behaviour (47). The prediction equation used in the present study to derive AEE is based on a small number of participants. However, it is derived from double-labelled water measurements, and as far as the authors know, this is the only equation based on free-living European children of the same age-group as in the present study (27). The accelerometers used in this study have also been shown to have good agreement with the double-labelled water technique comparing TEE_dlw_ to AEE, TEE or accelerometer counts (48).

## Conclusion

The WebDASC is both acceptable and feasible to use for collecting dietary data from schoolchildren aged 8–11 in a normal situation when children eat their usual packed lunches and during an intervention in which they are served an NND for school lunch and snacks. It performed better when estimating EI at group level and just as well when ranking individuals according to TEE when compared to other data-collecting methods in children. In the present study, recording accuracy was influenced by the child's gender, BMI, and illness.

More work needs to be done to optimize dietary data collection, for example, it should be investigated if the inclusion of a speech search could make up for the spelling competences of children (and adults), and if portion size estimation could be improved by using 3D images or other technology. Furthermore, it should be investigated how the web-based technology can help minimize misreporting. It looks as if the WebDASC will prove a very useful tool in future research of this kind.

## Appendix

**Table:** Menu plan weeks 9 and 12, served during the WebDASC validation study

Monday	Tuesday	Wednesday	Thursday	Friday
*Midmorning snack*	*Midmorning snack*	*Midmorning snack*	*Midmorning snack*	*Midmorning snack*
Apples and rye bread	Apples and rye bread	Skyr with muesli	Pears and rye bread	Pears and rye bread
*Lunch*	*Lunch*	*Lunch*	*Lunch*	*Lunch*
Pumpkin soup with roasted pumpkin seeds and skyr* dressing	Corned veal with root vegetables and horseradish sauce	Baked potato with crunchy spiced bread and mustard dressing	Baked Hake with breadcrumbs and corn salad with apples	Premade leftovers: Veal with pickles, hake with dill, pea puree and pumpkin soup
*Dessert*	*Dessert*	*Dessert*	*Dessert*	*Dessert*
Apple and pear slices		Apple cake		
*Afternoon snack*	*Afternoon snack*	*Afternoon snack*	*Afternoon snack*	*Afternoon snack*
Rye breadbar, salad leaves and dried cranberries	Rye breadbar, cabbage, and dried blueberries	Rye breadbar, carrots and dried cranberries	Rye breadbar, carrots and dried currants	Kamut-muesli bar, cabbage and dried cranberries

* *Skyr* is an Icelandic cultured dairy product, similar to strained yogurt.
